# Predictors of Grandparental Investment Decisions in Contemporary Europe: Biological Relatedness and Beyond

**DOI:** 10.1371/journal.pone.0084082

**Published:** 2014-01-08

**Authors:** David A. Coall, Sonja Hilbrand, Ralph Hertwig

**Affiliations:** 1 School of Medical Sciences, Edith Cowan University, Joondalup, Western Australia; 2 School of Psychiatry and Clinical Neurosciences, University of Western Australia, Crawley, Western Australia; 3 Department of Psychology, University of Basel, Basel, Switzerland; 4 Center for Adaptive Rationality, Max Planck Institute for Human Development, Berlin, Germany; London School of Hygiene and Tropical Medicine, United Kingdom

## Abstract

Across human cultures, grandparents make a valued contribution to the health of their families and communities. Moreover, evidence is gathering that grandparents have a positive impact on the development of grandchildren in contemporary industrialized societies. A broad range of factors that influence the likelihood grandparents will invest in their grandchildren has been explored by disciplines as diverse as sociology, economics, psychology and evolutionary biology. To progress toward an encompassing framework, this study will include biological relatedness between grandparents and grandchildren, a factor central to some discipline's theoretical frameworks (e.g., evolutionary biology), next to a wide range of other factors in an analysis of grandparental investment in contemporary Europe. This study draws on data collected in the Survey of Health, Ageing and Retirement in Europe from 11 European countries that included 22,967 grandparent–child dyads. Grandparents reported biological relatedness, and grandparental investment was measured as the frequency of informal childcare. Biological and non-biological grandparents differed significantly in a variety of individual, familial and area-level characteristics. Furthermore, many other economic, sociological, and psychological factors also influenced grandparental investment. When they were controlled, biological grandparents, relative to non-biological grandparents, were more likely to invest heavily, looking after their grandchildren *almost daily* or *weekly*. Paradoxically, however, they were also more likely to invest nothing at all. We discuss the methodological and theoretical implications of these findings across disciplines.

## Introduction

Across human cultures, grandparents and elders more generally are respected and valued contributors to the health of their families and communities. Disciplines as diverse as sociology, economics, psychology, and evolutionary biology and psychology have documented the impact grandparents have within families. Evidence from traditional societies shows that the presence of a grandparent can be as beneficial to child survival as, for instance, the introduction of a new water supply [1], [2]. In industrialized nations, the evidence is mounting that—especially in family environments with low resource availability—grandparents can buffer child development against difficult early environments [3], [4]. At the same time, however, millions of grandparents invest nothing—possibly because they are physically or emotionally remote or because they lack the necessary resources or inclination. All of the disciplines mentioned above seek to understand this variability, asking the questions: Why do (or do not) grandparents invest in their grandchildren? And what factors impact the levels of investment they provide?

With rapidly changing family structures in most industrialized nations and a concomitant change in the potential role of grandparents, grandparental investment is a burgeoning field of investigation. Yet although it cuts across several disciplines, there has to date been little cross-disciplinary research. Strong disciplinary barriers, misconceptions between disciplines, and exaggeration of disciplines' views have limited progress in the field [5]. While it is patent that each discipline makes valuable contributions to the study of grandparental investment, real progress in the field requires a comprehensive approach to grandparental investment. Against this background, we draw on an international database of older people to examine the contribution that evolutionary (biological), economic (macro- and micro-economic), demographic (fertility), sociological (region, intergenerational solidarity), and psychological (relationships, beliefs, and expectations) factors make to grandparents' inclination to invest in their grandchildren.

In the following we briefly review previous findings concerning factors impacting grandparental investment. Before we begin, let us clarify that with a few exceptions, it is impossible to confine a given variable or factor to a single theoretical perspective. Consider, for example, the variable used in this study: informal childcare provided by grandparents. Depending on the discipline's perspective, this variable can be described as intergenerational transfer (economic, evolutionary, and demographic perspectives), intergenerational solidarity (sociology), instrumental social support (psychology), or childcare (economics). Thus, one has to be careful in trying to categorize variables by discipline. Relatedly, a focus on one variable does not exclude, indeed often demands, the consideration of many other moderating variables. For instance, the focus on biological relatedness also necessitates the analysis of the impact of post-marital affiliations, lineage, sex and age of grandparents and grandchildren, family size, and characteristics of the environment (in this case, familial, economic, regional, and social [6], [7], [8], [9]).

### Does Biological Relatedness Impact Grandparental Investment?

Perhaps the most controversial and divisive issue between disciplines investigating grandparental investment is the role of biological relatedness [10]. The question other disciplines would ask of the evolutionary perspective is timely: Is the biological relationship between family members still relevant in contemporary societies? In industrialized societies, falling rates of marriage and high rates of divorce and remarriage have led to an increase in the proportion of non-kin, including grandparents, in many families. In 2009, for instance, the U.S. marriage rate was 6.8 per 1,000 people, with a divorce rate of 3.4 per 1,000 people [11]. After separation, 25% of women, who are more likely to have custody of their children, repartner within 2 years and remarry within 5 years [12]. Do the new, non-biological grandparents provide childcare equivalent to that provided by biological grandparents? Alternatively, do they invest less than biological grandparents, or are they wholly disengaged? To find out, we draw on an international database to examine the differences in informal childcare provided by grandparents who are or are not biologically related to their grandchildren.

Biological relatedness within a family matters. For instance, there is considerable evidence that closer biological relationships (and closer attachment) between children and family caregivers are associated with increased investment behaviors [13], [14], [15] and perceived obligations to those kin [16]. The impact of biological relatedness has been demonstrated in several lines of research. One, kin selection theory—the notion that inclusive fitness benefits stemming from the genetic relationship shared between grandparents and grandchildren lead grandparents to care for their grandchildren—attributes that behavior to the 25% shared biological relationship between grandparents and grandchildren. Recently, calls have been made to introduce genetic relatedness into cross-disciplinary studies for a more comprehensive understanding of grandparental investment [17]. We agree but also believe that the following question needs to be addressed: Can individuals' values such as filial expectations that are associated with grandparenthood [3], [5] compensate for the lack of biological relatedness?

Quality relationships with biological grandparents—associated with improved emotional health of grandchildren across nuclear, step-parent, and single-parent families [18]—cannot be taken for granted. Paternal grandparents may, for instance, become alienated after divorce, when the father typically leaves the household. Although maintaining quality contact with paternal grandparents after re-marriage and step-family formation appears to be beneficial to the behavioral adjustment and mental health of both grandparents and grandchildren [19], , we know little about the role non-biological grandparents (e.g., the step-father's parents) play in childcare and grandchildren's development. Circumstantial evidence supports the idea that—in analogy to step-parent families [14]—the relationship between step-grandparents and grandchildren is less advantageous to grandchildren than is their relationship with biological grandparents [22], [23], [24]. These preliminary findings are consistent with the thesis that step-grandparents are less inclined than biological grandparents to invest in their grandchildren. This thesis, however, has never been tested. Moreover, the datasets used to examine factors associated with grandparental investment are often limited to kin grandparent–grandchild dyads [25], [26], [27]. Our goal with the present study is to address the investment behavior of both biological and non-biological grandparents.

### Sex and Lineage Effects of Grandparents

Conceiving all grandparents, biological or non-biological, as equal investors would be naïve: Evidence from the sociological, psychological, and evolutionary literature suggests that different types of grandparents show different investment patterns [3], [28]. Perhaps, the most robust pattern is that maternal grandmothers invest the most, followed by maternal grandfathers, then paternal grandmothers, with paternal grandfathers investing the least. Different explanations exist. Sociological theorizing holds that women are kin-keepers, holding kin groups together [29], [30]. Similarly, according to family systems theory, it is the gatekeeper role of the parent (middle) generation that encourages (or not) the grandparent–grandchild relationship [16]. Thus, if the grandparent and parent are female (e.g., maternal grandmother), the bond between grandparent and grandchild will be stronger than if they were male (e.g., paternal grandfather), resulting in the pattern described. Evolutionary perspectives attribute this association between grandparent type and investment to sex-specific reproductive strategies and paternity uncertainty (see Table 1 in [3]). Whereas women are 100% certain who their children are, males are generally less than 100% certain that they are the biological father of their children. Grandparents with higher levels of certainty of their biological relationship to their grandchildren (maternal grandparents) invest more than those with lower levels of certainty (paternal grandparents; see [26], [31], [32]). Finally, from a psychological perspective, it has been proposed that this pattern may result from the well-known differences in age and life expectancy between grandparent types [33]. These different perspectives make similar and largely compatible predictions [34], [35] even though they focus on different levels of explanation (i.e., mechanistic versus adaptationist).

**Table 1:** Individual, familial and macro-economic characteristics of biological and non-biological grandparents^a^.

	Biological (*n* = 20,710)			Non-biological (*n* = 2257)			
	Mean (%^b^)	SD^c^	*n*	Mean (%^b^)	SD^c^	*n*	*p*
Almost daily childcare	8.8		1819	3.8		85	***
Almost weekly childcare	15.5		3210	11.4		256	**
Almost monthly childcare	10.6		2186	12.9		289	*
Less often childcare	15.0		3103	19.9		448	**
Never childcare	50.1		10356	52.0		1170	*
Grandparent sex (female)	57.6		11934	45.4		1025	***
Grandparent lineage (maternal)	50.8		10523	50.5		1140	
Filial expectations	3.8	0.8	13743	3.6	0.8	1600	***
Distance to (grand)child	4.7	1.9	20681	5.2	2.0	2230	***
Number of children	2.6	0.9	20710	3.0	0.9	2257	***
Number of grandchildren	3.9	2.6	20710	4.2	3.0	2257	*
Grandparent's age	68.5	9.8	20702	63.8	9.1	2257	***
Grandparent's health	3.5	0.9	10131	3.7	1.0	1130	***
Conflict with children (high)	28.9		3785	29.9		451	
Conflict about grandchildren's upbringing (high)	12.8		1626	8.7		124	***
Savings (in euro)	19800	643656	7722	35498	196906	1106	
Grandparent's education	4.4	4.9	18815	4.9	4.7	2170	***
Grandparent employed (yes)	30.2		4890	29.4		541	
Grandparent has a partner (yes)	61.0		12641	78.0		1761	***
Age of child	36.8	9.7	20533	32.3	10.6	2240	***
Education of child	5.7	4.8	19609	6.0	4.5	2084	***
Child employed (yes)	78.9		16059	72.8		1517	***
Child has a partner (yes)	75.4		14857	74.3		1397	
Age of youngest grandchild	10.1	8.5	12654	8.7	8.1	1143	***
Fertility rates	1.5	0.2	20710	1.7	0.2	2257	***
Regions (north/central)	60.9		12617	87.1		1966	***

^a^ Statistical comparisons between biological and non-biological grandparents were made using chi-square or Mann–Whitney U tests.

^b^ percentage is shown for categorical variables.

^c^ standard deviation is absent for categorical variables.

*p<.05. ** *p*<.01. *** *p*<.001.

### Numerous Non-Biological Factors Drive Investment Decisions

The investment decisions made by biological and non-biological grandparents are of course not necessarily due to differences in biological relatedness. Other factors may also impact investment. For instance, a non-biological grandparent whose child has divorced and remarried may be older or less healthy, have more children and grandchildren, have fewer resources to invest, feel less obligation to the family, or live further away from his/her grandchildren. Such factors would affect the availability of grandparental resources and may be more pronounced in non-biological grandparents. Indeed, this is where the predictions of evolutionary models diverge from those of economic and sociological perspectives [5] such as the *rational grandparent* model [28]. This model holds that grandparental investment is indifferent to biological relatedness and that grandparents will preferentially invest in those descendants who are most likely to reciprocate in the future.

Next to these individual characteristics, it is also important to consider macroeconomic factors potentially impacting grandparental investment, such as the interaction between welfare-state systems and grandparental investment. Using the Survey of Health, Ageing and Retirement (SHARE), one study found a north–south gradient in grandparental childcare [36]. Danish, Dutch, French, and Swedish grandparents were more likely to provide any care for their grandchildren but were less likely to provide it regularly. Austrian, German, and Swiss grandparents showed average levels of both any care and regular care. In Greece, Italy, and Spain, grandparents were less likely to provide any care, but when they did, it was more likely to be regular. The authors suggested that the higher availability of state-provided childcare in northern European countries promotes maternal employment, meaning that grandparents are needed to supplement institutional care. Conversely, in Mediterranean countries, where state-run childcare is less widespread and more expensive, levels of maternal employment are lower. If the mother is employed, however, grandparents become regular childcare providers [37], [38]. Coall and Hertwig [4] investigated the implications of this association further and found that low levels of regular care and high levels of any care were strongly associated with higher fertility rates across Europe. Thus, regional differences in state-provided childcare and female employment rates, which may be reflected in national fertility rates, also have consequences for the grandparental investment in contemporary industrialized nations. In this study, we will use national fertility rates as a course proxy for these macroeconomic factors.

Of course, not all differences in grandparental investment between regions of Europe are associated with welfare state regimes, the role of women in the workforce, and thus national fertility rates. Regional preferences, independent of macro-economic factors, are likely to also influence grandparental investment. Kaptijn and Thomese [39] highlighted the Netherlands as an example of this: the joint presence of parental preferences for grandparents as childcare providers and high availability of state-funded childcare in the Netherlands suggests that, in some circumstances, regional preferences (values) have the power to outweigh macro-economic influences. In the present study, regions of Europe (north/central and south/central) will be used to examine the potential influence of regional differences on grandparental investment across Europe.

In sum, we investigate three issues: (1) Does biological relatedness influence grandparental investment patterns in contemporary Europe? (2) Do various non-biological factors—that is, age, health, sex, lineage, distance, family size, employment, marital status, family obligations and conflict, geographic regions, and fertility rates—vary between biological and non-biological grandparents and influence their investment decisions? (3) Assuming that non-biological and biological grandparents differ systematically on non-biological factors, do these differences fully account for differential investment patterns of non-biological and biological grandparents—or is biological relatedness an indispensable explanatory factor in contemporary Europe? In order to study these questions, we drew on data from the large-scale international dataset collected in the context of the Survey of Health, Ageing and Retirement in Europe (SHARE).

## Methods

### Sample

Our empirical analysis was based on the first wave of the multidisciplinary SHARE project, which was conducted in 2004. Data were collected across 12 countries from a representative sample of participants aged 50 or older and their partners. A computer-assisted interview and paper-and-pencil questionnaire covered aging-related topics such as health, social and family networks, and financial situation (for details, see [40]). In the present investigation, the sample was restricted to European respondents (generation 1: grandparents; G1) from Austria, Belgium, Denmark, France, Germany, Greece, Italy, Netherlands, Spain, Sweden, and Switzerland who had either biological or non-biological children (generation 2: children; G2) (to a maximum of four children) and at least one grandchild (generation 3: grandchildren; G3) (not older than 14 years). On average, each respondent (G1) had 2.7 children (G2) and 4.0 grandchildren (G3). To examine each grandparent–child relationship (G1–G2), the dataset was transformed into 22,967 observations representing 12,959 grandmother–child dyads (56.4%) and 10,008 grandfather–child dyads (43.6%). Of the total dyads, 2257 were non-biological (9.8%).

It is important to note that we explore grandparents' (G1) investment in grandchildren (G3) through the grandparent–child (G1–G2) dyad. As such, most of the variables explored, including biological relatedness, reflect the grandparent–child relationship. Information on the sex of grandchildren (G3) and their biological relationship to their parents (G2) were not available in the SHARE dataset. A detailed overview of the descriptive data is available as Table S1 in File S1.

### Measures

*Grandparental investment*, the dependent variable, was measured by integrating responses to two questions. First, grandparents (G1) were asked whether they had looked after their grandchildren (G3) in the past 12 months, with the response categories ‘Yes’ and ‘No’. Second, those participants (G1) who answered positively were then asked, independently of their spouse, how often they had looked after their grandchildren (G3) without the presence of the parents (G2) in the last 12 months. This question is of particular value as a measure of investment, because looking after grandchildren without the presence of the parents provides resources to the parents (G2) [41] and has opportunity costs for the grandparents (G1) [42]. Thus, it is a clear measure of grandparental investment in terms of the instrumental support or tangible benefits provided to the family. The answers to the two questions were merged to produce a 5-point ranking scale of grandparental investment: almost daily (5), almost weekly (4), almost monthly (3), less often (2), and never (1).

The *biological* versus *non-biological grandparent* variable was determined from the following question addressed to grandparents (G1): “Is this child a natural child/Are all these children natural children of your own [and your current spouse or partner]”? From the responses to this question, grandparents were categorized as being either biologically related to all or none of the children (G2) they were questioned about. Parents (G1) who are not biologically related to their children (G2) cannot, by extension, be related to their grandchildren (G3) by those children. This process established the biological relatedness of each grandparent–child dyad (G1–G2). Grandparents' answers were recoded into 0 (non-biological grandparent) or 1 (biological grandparent).

Grandparent's *sex* was coded as 0 (grandfather) or 1 (grandmother). The sex of the child (G2) was used to compute the *lineage* variable that denotes for each grandparent–child dyad whether a grandparent is paternal (0) or maternal (1). Assuming that grandchildren (G3) under the age of 14 usually live with their parents (G2), *distance* to each (grand)child was measured on a 9-point scale, ranging from living “in the same household” to “more than 500 kilometers away, abroad.” There was no question directly probing how far grandparents lived from their grandchildren. *Number of children* (G2) *and grandchildren* (G3) was directly extracted from the original SHARE variables. *Age* of grandparents, children, and grandchildren was computed by subtracting the year of birth from the year that the interview was conducted. The 5-point scale of grandparental *health* was reverse coded to range from 1 (very bad) to 5 (very good).

The variable *filial expectations* subsumed four items probing grandparents' endorsement of statements relating to family obligations and grandparenting roles: (1) “Parents' duty is to do their best for their children even at the expense of their own well-being”; (2) “Grandparents' duty is to be there for grandchildren in cases of difficulty (such as divorce of parents or illness)”; (3) “Grandparents' duty is to contribute towards the economic security of grandchildren and their families”; and (4) “Grandparents' duty is to help grandchildren's parents in looking after young grandchildren.” For each grandparent, a composite score was calculated by averaging the four responses (given on a 5-point scale that we reverse coded to range from 1 =  “very low” to 5 =  “very high”). The scale had good internal consistency, with a Cronbach's alpha coefficient of .78.

Two questions concerned *conflicts with children* (G2). The first, general question read: “There are sometimes important questions about which we have a disagreement with persons close to us, and which therefore may lead to conflicts. Please tell us how often, if at all, you experience conflict with each of the following persons: d) children” (the other options are not relevant to the present analysis). The second, more specific question asked about conflicts over the *upbringing of grandchildren*: “How often do you experience conflicts with your children or children-in-law over the education and bringing up of your grandchild(ren)?”. The four response alternatives to each question were dichotomized into two groups: *low* (“rarely,” “never”) and *high* (“often,” “sometimes”) conflict.

Bank *savings* in euro was used as a proxy for grandparents' financial status. Concerning grandparents' (G1) and children's (G2) *education*, SHARE provides standard coding for international comparisons (ISCED-97), where a higher category number (1–19) indicates a higher educational level. Data on the working and partner status of grandparents (G1) and children (G2) were dichotomized into *gainfully working* or not and *living with a partner* or not. The categorical variable *regions* was computed with reference to the findings of Hank and Buber [36], who found a north–south gradient in grandparental childcare in Europe using the SHARE database. Our variable therefore distinguishes between the *north/central* (1) region (Sweden, Denmark, France, Belgium, Netherlands, and Germany) and the *south/central* (0) region of Europe (Austria, Switzerland, Italy, Spain, and Greece). Finally, *fertility rates* from 2004 were obtained electronically from the Population Reference Bureau [43] and added to the database manually for each country. These figures show the average total number of children a woman will have at current age-specific birth rates. Compared with other regions of the world, the fertility rates of all countries in our sample are low, ranging from 1.32 to 1.92. However, there is a gradient reflecting the north–south axis through Europe, with the lowest fertility rates in Italy and Greece and the highest in France, Denmark, and Sweden (for details, see Table S1 in File S1).

### Data Analysis

The data analysis proceeded in four main steps. First, we analyzed whether biological and non-biological grandparents differed in levels of grandparental investment as well as in various non-biological factors (see Table 1). Categorical variables were analyzed with chi-square tests (with Yates' correction for continuity) and continuous variables with Mann–Whitney U tests. Second, we additionally used Spearman correlations to analyze whether grandparents' non-biological characteristics varied according to their level of investment and therefore were identified as confounders (see Table S3 in File S1). Third, we used multinomial logistic regression to examine whether any effect of biological relatedness (or lack thereof) on the level of grandparental investment could be explained by variation in non-biological grandparental characteristics. Grandparental investment levels were used as the dependent variable and the characteristics as covariates (see Table 2; Table S6 in File S1). Accounting for the clustered structure of the data, we used a *household identifier* provided by SHARE to control for grandparent–child dyads (G1–G2) originating from the same grandparents. The household identifier, scrambling coding of the country, household and personal record number for each grandparent (13 digits), was sorted in ascending order and included as control variable in the regression model. Geographic clusters were controlled by the variable *regions*.

**Table 2:** Odds ratios (Exp[B]) and significance levels for each grandparental investment level: results of a multinomial logistic regression analysis.

	Almost daily childcare		Almost weekly childcare		Almost monthly childcare		Less often childcare	
	Exp(B)	*p*	Exp(B)	*p*	Exp(B)	*p*	Exp(B)	*p*
Biological grandparent (yes)	1.51	*	1.57	*	0.98		1.10	
Grandparent sex (female)	1.24		1.22		1.31	*	1.29	*
Grandparent lineage (maternal)	1.54	**	1.06		0.83		1.07	
Filial expectations	1.79	***	1.24	**	1.46	***	1.09	
Distance to (grand)child	0.71	***	0.79	***	0.98		1.14	***
Number of children	0.71	**	0.97		1.11		1.20	*
Number of grandchildren	1.08	*	1.04		1.00		0.98	
Grandparent's age	0.92	***	0.93	***	0.93	***	0.96	***
Grandparent's health	0.83	*	1.18	**	1.10		1.23	***
Conflict about grandchildren's upbringing (high)	1.18		0.86		0.87		0.84	
Grandparent's education	1.01		1.09	***	1.09	**	1.05	
Grandparent has a partner (yes)	1.79	***	1.38	**	1.14		0.93	
Age of child	0.93	***	0.94	***	0.97	*	0.97	*
Education of child	1.09	**	1.00		0.93	*	0.97	
Child employed (yes)	1.95	***	1.08		1.37	*	1.05	
Age of youngest grandchild	0.91	***	0.92	***	0.90	***	0.93	***
Fertility rates	0.13	**	0.81		4.02	**	5.41	***
Regions (north/central)	0.43	**	1.13		1.66	*	1.03	
Household identifier	0.97		0.96		0.99		1.00	

*p<.05. ** *p*<.01. *** *p*<.001.

Multinomial logistic regression allows us to analyze each level of an ordinal outcome variable relative to the reference level. The reference level in this study was *no investment*. For each of the remaining investment levels (*almost daily*, *almost weekly*, *almost monthly*, and *less often*), the variance explained by each covariate was tested for significance in relation to *no investment* (odds ratio). Furthermore, the estimated probabilities for each investment level can be calculated and saved as a new variable in the database. Only true confounders were included in the regression model, that is, those covariates that are significantly associated with both biological relatedness and grandparental investment, and that therefore potentially account for the variance between the two variables (see Table S3 in File S1). The one exception was the covariate *lineage* (and therefore sex of child). Statistically, there was no association with biological relatedness, which is easily explained: a child's sex cannot be expected to be dependent on whether or not the parent is a biological relative. However, there was a strong association with investment, as expected from several theoretical perspectives. This important covariate was therefore included in the final model. The assumptions for multinomial logistic regression, such as sample size, multicollinearity, and outliers were met, and the potential mediator effect of age on health was examined (see Table S4 in File S1).

Taking advantage of the multinational SHARE database, we examined the independent influence of geographic regions and fertility rates separately. Both covariates were found to be independent predictors of grandparental investment and were used in subsequent analyses. Further information on the use of these country-specific parameters is included in Table S5 in File S1. Before running the final analysis, we tested the results for robustness (see Tables S7, S8, and S9 in File S1). In addition, we examined whether grandparents who looked after their grandchildren on a daily basis were in fact probably substitute parents, as SHARE does not provide information about custodial care (Table S2 in File S1).

**Figure 1:**
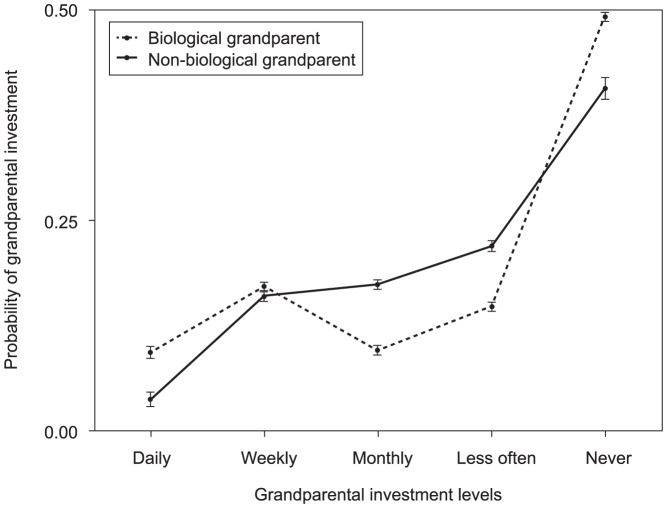
Probability of grandparental investment. Probability of grandparental investment across grandparental investment frequency and biological relatedness showing means and the standard error of the mean.

As the final step of the analysis, we conducted a mixed between-within subjects analysis of variance (Figure 1), and tested the effect of being a biological versus non-biological grandparent across all investment levels, including no investment, instead of relative to it (Figure 1). Furthermore, this procedure allowed us to evaluate the mean differences and to test for interactions between biological and non-biological grandparent variables and investment levels. The dependent variable *probability of grandparental investment* includes the influence of the true confounders, as estimated probabilities for each investment level were saved from the previous multinomial logistic regression procedure.

## Results

### Does Biological Relatedness Influence Grandparental Investment?

First and foremost, were there any differences in the investment of biological and non-biological grandparents? Yes, there were. Specifically, the proportion of biological grandparents reporting investment on a daily basis was more than double that of non-biological grandparents (8.8% versus 3.8%, see Table 1). Likewise, more biological than non-biological grandparents looked after their grandchildren on a weekly basis (15.5% versus 11.4%). However, more non-biological than biological grandparents reported investment on a monthly basis or less often, and around 50% of both groups reported no investment at all. These differences in investment could be due to non-biological factors, biological relatedness, or a combination of both. The following analyses aim to determine the relative contribution of these factors.

### Which Factors Contribute to Grandparents' Investment Decisions?

We examined on which non-biological characteristics grandparents, their children and grandchildren differed as a function of whether the grandparent was biologically related to the grandchild's parent (G2). Table 1 lists the results. In fact, the majority of characteristics varied significantly between biological and non-biological grandparents. To begin with, a significantly larger proportion of biological than non-biological grandparents in the sample were grandmothers; however, there was no difference in the proportion of grandparents who were maternal versus paternal. Next, biological grandparents felt significantly more obliged to help their family than did non-biological grandparents. Furthermore, biological grandparents lived closer and had fewer children and grandchildren than did non-biological grandparents. A higher sense of duty, closer proximity, and fewer recipients of their investment could all contribute to making biological grandparents higher investors.

However, other differences are likely to deplete the resources of biological grandparents or make them less inclined to invest, relative to non-biological grandparents. Specifically, biological grandparents were older, reported poorer health and lower educational attainment, and were less likely to have a partner than non-biological grandparents. Furthermore, biological grandparents reported more conflicts with their children (G2) about the upbringing of their grandchildren than did non-biological grandparents; however, there was no difference in conflicts with children generally. The biological grandparents in the sample were less likely to be from the north/central region of Europe and exhibited the associated lower fertility rates. Some country-specific structural and regional factors that may be reflected in fertility rates and geographic borders therefore seem to affect the chance of becoming a non-biological grandparent, which may further impact the level of grandparental investment. In terms of financial status, there were no differences in the amount of savings grandparents had or their likelihood of employment. Last but not least, there were some differences between the children of biological and non-biological grandparents. The children of biological grandparents were older, had lower educational attainment, and were more likely to be employed. There was no difference in the proportion of children having a partner.

In sum, numerous significant differences between biological and non-biological grandparents were observed. Some of these differences are likely to favor higher investments by biological than non-biological grandparents (e.g., sense of obligation, smaller distances), whereas others impede higher investments (e.g., older age, poorer health). In light of these results, we next examined which grandparental characteristics, across biological and non-biological grandparents together, were significantly associated with high grandparental investment (Table S3 in File S1). In combination with the initial analysis (Table 1), we thus established true confounders of the relationship between biological relatedness and grandparental investment by identifying those characteristics associated with both variables. Before turning to this analysis, we examined whether there was any indication that grandparents who looked after their grandchildren on a daily basis were substitute parents. There was no evidence that this was the case (Table S2 in File S1), suggesting that daily investment exacts opportunity costs for the grandparents and is therefore a genuine measure of investment.

### Do Non-Biological Factors Account for the Biological Relatedness Effect?

All true confounders plus lineage and a variable controlling for households were entered into a multinomial logistic regression to determine whether the association between biological relatedness and grandparental investment was an independent effect or could be accounted for by one or several grandparent characteristics. Table 2 shows which of the covariates significantly explained variance in each of the grandparental investment levels relative to the reference level (no investment). Odds ratios and significance levels of covariates are given for each investment level. Table S6 in File S1 presents more statistical details.

Biological grandparents were 1.5 times as likely as non-biological grandparents to invest on a daily (*p*<.04) or weekly basis (*p*<.02), relative to non-investors. There was no significant difference between these two groups at the level of monthly or less frequent investment. The variance explained by the total model was high, with a Nagelkerke's *R* ^2^ of 44.5%.

Key findings with respect to the covariates include consistent positive associations between filial expectations and the probability of grandparental investment on a daily, weekly, and monthly basis. Moreover, younger grandparents were more likely to invest, and younger children and grandchildren were more likely to receive investment, across all levels of investment. For other variables, the association changed with investment level. Children of working parents (G2) were more likely to receive grandparental care on a daily and monthly basis. Greater geographical distance to grandchildren was associated with lower investment on a daily and weekly level, but higher investment on a less frequent basis. Having more children reduced the likelihood of daily investment, but increased the probability of investment on a less frequent basis. Interestingly, having more grandchildren *increased* the likelihood of daily investment. Higher fertility rates were associated with a significantly decreased likelihood of grandparents looking after their grandchildren on a daily basis, but a strongly increased probability of grandparental care on a monthly and less frequent basis. Living in north/central Europe significantly decreased the chance of daily investment, but increased the chance of investment on a monthly basis. Both variables indicate more frequent grandparental investment in the southern countries, where fertility rates are lower than in the north. These results are in line with the results of Hank and Buber [36], who found that grandparental investment is prevalent across Europe, but more intense in the southern countries.

We tested the robustness of these results by using different statistical methods and altering the categorization of grandparental investment. Similar results emerged when we used binary logistic regression and dichotomized the investment variable into high (almost daily/weekly) and low (almost monthly/less often/never) investment. Moreover, both multinomial and binary logistic regression still produced similar results when all the non-investors (50.3% of the sample) were excluded, suggesting that these are robust effects. The results of these additional analyses are available in Tables S7, S8, and S9 in File S1.

### Grandparental Paradox: Biological Grandparents Invest Heavily or Not at All

Finally, we examined the mean differences between biological and non-biological grandparents in the estimated probabilities of grandparental investment levels. Figure 1 plots the results by investment levels and grandparental group. When interpreting these results, it is important to bear in mind that this analysis measures mean differences in the probability of grandparental investment, which is not relative to any investment level (as was the case in the multinomial logistic regression). The most striking result is that biological grandparents were significantly more likely than non-biological grandparents to invest at both extremes of the investment spectrum. Biological grandparents were more likely to invest heavily, looking after their grandchildren *almost daily* or *weekly*, but they were also more likely to invest nothing at all. Non-biological grandparents showed a higher probability of investing *almost monthly* or *less often*.

To determine whether the different investment inclinations between biological and non-biological grandparents were significant, we investigated the interaction. The interaction term was significant, showing that the level of investment depended strongly on whether or not the grandparent was biological (Wilks' lambda  = .90, *F*(4, 3813) = 106.69, *p*<.0005, partial η^2^ = .10). In this model, the main effect of biological versus non-biological grandparent remained significant (*F*(1, 3816) = 277.25, *p*<.0005, partial η^2^ = .07), as did the main effect of investment level (Wilks' lambda  = .50, *F*(4, 3813) = 983.35, *p*<.0005, partial η^2^ = .50).

## Discussion

The present investigation is the first to show that the biological relationship between grandparents and grandchildren contributes to variation in grandparental investment in modern European societies, independent of a wide range of non-biological factors. Biological grandparents were more likely than non-biological grandparents to make high investments in their grandchildren. This evidence supports kin selection theory [44], which was previously untested in grandparents. Paradoxically, biological grandparents were also more likely not to invest at all. To our knowledge, this is a unique finding in the grandparental investment literature. We speculate on the potential causes of this association below. Equally important, however, is the finding that a range of non-biological factors impacted grandparents' investment decisions. This finding highlights the need for an encompassing approach in this field: social, economic, psychological, and evolutionary factors all play a role in explaining the variance in grandparental investment behaviors.

### How do Biological and Non-Biological Grandparents Differ?

Central to understanding why biological and non-biological grandparents invest differently in their grandchildren are the dimensions on which they differ (Table 1). Many factors previously associated with increased investment are also correlated with being biologically related to grandchildren [45], [46]. Specifically, biological grandparents were more likely to be female, felt more duty to their family, lived closer, had fewer children and grandchildren, and their children were more likely to be employed. On the other hand, biological grandparents also had characteristics commonly associated with reduced investment: They were less healthy and older, as were their children and grandchildren. Moreover, they were less likely to have a partner, and—perhaps because they do invest more—had more conflicts with their children about how their grandchildren are brought up. At the macro-economic level, biological grandparents were more likely to be from south/central European nations with lower average fertility rates. Although these factors accounted for more than 40% of the variance in grandparental investment, they did not fully account for the higher investment by biological grandparents. This relationship was robust to alternate statistical methods and the dichotomization of grandparental childcare into “high” and “low” investment categories [25], [36]. Our findings thus suggest that biological relatedness between grandparents and their children remains an important predictor of grandparental investment in contemporary industrialized European societies.

### The Two Faces of Investment by Biological Grandparents

Studies of grandparental investment consistently focus on grandparents who do invest. In light of this focus, it is striking that approximately 50% of biological grandparents did not invest at all, at least not in the form of informal childcare (Figure 1). At this point we can only speculate about the reasons. Biological grandparents may be more likely to experience conflict in the family and thus estrangement. Consistent with this, biological grandparents were significantly more likely than non-biological grandparents to report conflicts about the upbringing of their grandchildren (Table 1). It is also likely that some biological grandparents provide resources other than time. They may be financial—in the form of an inheritance or help with the costs of education—or they may take the form of emotional support. All of these resources are valuable aspects of intergenerational solidarity that we did not consider in the present analysis.

Our results have implications for understanding the “units” in which grandparents invest. Having more children strongly decreases *almost daily* investment, whereas having more grandchildren independently increases investment. This finding suggests that it is specifically the number of family units between which grandparents split their investment that reduces investment, rather than the absolute number of grandchildren. Consistent with this interpretation, in a Swiss study of grandparent–grandchild relationships, Coall and colleagues [45] found that earlier reproductive scheduling and having more children and grandchildren were associated with reduced grandparental investment across a range of measures. The present study confirms that grandparental investment, like parental investment in humans [47], is strongly associated with reproductive scheduling.

### Theoretical Implications

Next, we discuss theoretical implications that our findings have. First, the finding that the biological relationship between grandparents and grandchildren is an independent predictor of high grandparental investment, even in contemporary European nations, is consistent with kin selection theory [44]. The impact of biological relatedness is often seen as incompatible with sociological and economic models of parental and grandparental investment [5], [28]. In these models, investment is often assumed to preferentially flow to those grandchildren (and their parents) who are more likely to reciprocate in times of need. If, however, non-biologically related individuals are less likely to reciprocate in the future, which an evolutionary perspective would suggest, our findings may simultaneously support the predictions of the sociological, economic, and evolutionary accounts. Reciprocal altruism, which is most often conceptualized as exchanges between unrelated individuals, is likely to have originally evolved in close kin groups. The psychological traits that maintain a system of reciprocity in humans (e.g., guilt, trust, sympathy, gratitude [48]) are likely to be stronger between close kin and to promote kin as less risky partners with whom to reciprocate [49]. Similarly, just as they are proposed to do in parent-child relationships [14], quality grandparent-grandchild attachment relationships may provide a crucial proximate mechanism whereby grandparents identify and preferentially care for biological grandchildren [26], [50]. Indeed, the many non-biological grandparents who *do* invest may do so because of particularly harmonious relationships between family members. It is therefore likely that investment in biological grandchildren improves inclusive fitness and is simultaneously more likely to be reciprocated. Consequently, our findings are not necessarily at odds with economic or sociological accounts of grandparental investment.

Second, there are also challenges to all these theoretical perspectives. If biological relatedness or the expected reciprocation are central, why is it that so many grandparents, both biological and non-biological, do not invest? Obviously, these theories are not designed to explain or predict a lack of investment. Unfortunately, by definition, large-scale databases provide less information on respondents who do not invest, and therefore little is known currently about why grandparents do not invest.

Third, our investigation found no evidence for some predictors of grandparental investment that are commonly found. The most obvious of these is the effect of grandmothers investing more than grandfathers, and maternal grandparents investing more than paternal grandparents [2], [26], a finding that has been previously identified in this database when the focus was on biological grandparent–grandchild dyads [25]. In the current analysis, significantly more biological grandparents were grandmothers, which may reflect divorce and remarriage patterns, and investment by grandmothers in more certain kin [51]. These findings raise questions about the boundary conditions of patterns of grandparental investment by sex and lineage.

### Limitations

Our investigation has several limitations. Among them, the main one is that the biological relatedness variable focuses on whether the grandparent is related to his/her children (and, by extension, to his/her grandchildren). If a grandparent divorces and re-marries, he/she may then have non-biological children. Similarly, if the grandparent has children via adoption, they will be non-biological. However, we do not have information on the biological relationship between the second (children) and third (grandchildren) generations. That is to say, we cannot take into account divorce or adoption in the parents' generation (G2). This limitation means that our estimate of biological relatedness is overestimated and that larger effects of biological relatedness may be present.

A second limitation is the (relative) scarcity of individual-level information. On the one hand, we were able to draw on extensive information about the grandparents: their tangible investments in the form of informal childcare, their children's employment, family structure, conflicts within the family, and obligations towards the family. On the other hand, more information on other contacts between grandparents and grandchildren, socio-economic resources, and the demands on them from other family members would have improved the analysis [52]. Unfortunately, the SHARE database does not include information on other types of investment, such as financial support, which may reveal different patterns of investment [53]. Also, because we were unable to establish whether grandparents have both biological and non-biological children, we were not able to conduct within-family comparisons. Nonetheless, the fact that we included numerous control variables minimizes the risk that this finding is spurious.

A third limitation is the blunt measure our regions variable provides. Our aim was to adjust for potential regional differences in grandparental investment patterns. There is, however, a multitude of unmeasured cultural factors that impact grandparental investment decisions and may account for further variance in our models. Cross-cultural analyses show that culture-specific differences impact grandparental investment patterns [17]. Future research could use large datasets such as SHARE complemented with diverse measures of cultural differences to examine their impact on investment.

Last but least, let us emphasize that our investigation concerned quantity of investment, not its consequence. We cannot determine whether biological or non-biological grandparental investments are more *beneficial* to grandchildren, and the patterns of available evidence do not permit simple conclusions. Interventions designed to promote interactions between unrelated older people (≥60 years) and adolescents—not dissimilar to contact between grandparents and grandchildren—have been shown to have cognitive or health benefits for both generations [54]. However, under some conditions, purportedly biologically related grandparents (but see [17]) can decrease the probability of their grandchildren surviving (e.g., paternal grandmothers; [55], [56]). Conversely, in low-resource family environments, grandfathers can fill crucial roles within the family [57]. To reiterate: We found that, independent of a range of likely confounding factors, non-biological grandparents are less likely to invest intensively in their grandchildren—the consequences of these investments, or lack thereof, for grandchildren remain open.

## Conclusion

Across human societies, both biologically and non-biologically related individuals contribute to the survival and development of subsequent generations. As fertility rates fall and divorce and remarriage rates rise, the proportion of non-biologically related family members in western families is increasing. Unfortunately for parents and their children, having more grandparents to call upon in theory does not mean more support in practice: Non-biological grandparents are less likely to provide high levels of informal childcare. Data from this multi-national investigation of European societies are thus still consistent with a crucial theoretical underpinning of the modern evolutionary synthesis, namely that biological relatedness is a predictor of investment behavior [44]. Paradoxically, we also found that biological relatedness is associated with an increased risk of providing no grandparental investment at all. Crucially, our study highlights the necessity of a comprehensive framework of grandparental investment including sociological, economic, psychological, and evolutionary measures and concepts.

## Supporting Information

File S1 Contains supporting information explaining the data analysis in more detail and additional analyses to test the robustness of the initial multinomial logistic regression predicting grandparental investment. **Table S1,** Descriptive data; **Table S2,** Hours of grandparental investment for the almost daily level; **Table S3,** True confounders between biological relatedness and grandparental investment; **Table S4,** Mediation effect of grandparent's age by health on grandparental investment; **Table S5,** Independent effects of fertility rates and regions of Europe; **Table S6,** Results for each investment level from the multinomial logistic regression; **Table S7,** Binary logistic regression for the entire sample including non-investors; **Table S8,** Multinomial logistic regression for each grandparental investment level excluding non-investors; **Table S9,** Binary logistic regression excluding non-investors.
(DOCX)
