# Supplementary material for: Predictors of Grandparental Investment Decisions in Contemporary Europe: Biological Relatedness and Beyond
Source: PLoS One. 2014 Jan 8;9(1):e84082. doi: 10.1371/journal.pone.0084082 (PMC3885520; doi:10.1371/journal.pone.0084082)
Supplement: File S1 — Contains supporting information explaining the data analysis in more detail and additional analyses to test the robustness of the initial multinomial logistic regression predicting grandparental investment. Table S1, Descriptive data; Table S2, Hours of grandparental investment for the almost daily level; Table S3, True confounders between biological relatedness and grandparental investment; Table S4, Mediation effect of grandparent's age by health on grandparental investment; Table S5, Independent effects of fertility rates and regions of Europe; Table S6, Results for each investment level from the multinomial logistic regression; Table S7, Binary logistic regression for the entire sample including non-investors; Table S8, Multinomial logistic regression for each grandparental investment level excluding non-investors; Table S9, Binary logistic regression excluding non-investors. (DOCX) [file pone.0084082.s001.docx]

**Supporting Information**

for

**Predictors of Grandparental Investment Decisions in Contemporary Europe:**

**Biological Relatedness and Beyond**

**David A. Coall^1,2*^, Sonja Hilbrand^3^, Ralph Hertwig^4^**

**1** School of Medical Sciences, Edith Cowan University, Joondalup, Western Australia

**2** School of Psychiatry and Clinical Neurosciences, University of Western Australia, Crawley, Western Australia

**3** Department of Psychology, University of Basel, Basel, Switzerland

**4** Center for Adaptive Rationality, Max Planck Institute for Human Development, Berlin, Germany

* Correspondence concerning this article should be addressed to David A Coall ([d.coall@ecu.edu.au](mailto:d.coall@ecu.edu.au))

This file provides Supporting Information explaining the data analysis in more detail. First, Table S1 presents descriptive data for the dependent and independent variables and covariates used in the study. Second, we investigate whether grandparents who look after their grandchildren on a daily basis are in fact probably substitute parents—SHARE does not provide this information (Table S2). Third, Table S3 lists true confounders identified to be significantly associated with both biological relatedness and grandparental investment. Fourth, we explore the potential mediation effect of age on health (Table S4). Fifth, Table S5 evaluates the independent effects of fertility rates and geographic regions on grandparental investment. Sixth, Table S6 displays additional details of the main multinomial logistic regressions conducted. Last, we tested the robustness of the initial analysis by re-running the analyses using different methods and altering the measurement of grandparental investment (Tables S7, S8, and S9).

**Table S1.** Descriptive data for dependent, independent, and covariates used in this study.

| **Variable** | **Categories** | **Mean (%^a^)** | **SD^b^** | ***N*** |
| --- | --- | --- | --- | --- |
| **Grandparents** |  |  |  |  |
| Investment | Almost daily childcare | 8.3 |  | 1904 |
|  | Almost weekly childcare | 15.1 |  | 3466 |
|  | Almost monthly childcare | 10.8 |  | 2475 |
|  | Less often childcare | 15.5 |  | 3551 |
|  | Never childcare | 50.3 |  | 11526 |
| Biological grandparent | Yes | 90.2 |  | 20710 |
|  | No | 9.8 |  | 2257 |
| Grandparent sex | Female | 56.4 |  | 12959 |
|  | Male | 43.6 |  | 10008 |
| Grandparent lineage | Maternal | 50.8 |  | 11663 |
|  | Paternal | 49.2 |  | 11304 |
| Filial expectations |  | 3.8 | 0.8 | 15343 |
| Distance to (grand)child |  | 4.7 | 1.9 | 22919 |
| Number of children |  | 2.7 | 0.9 | 22967 |
| Number of grandchildren |  | 4.0 | 2.6 | 22967 |
| Grandparent’s age |  | 68.7 | 9.8 | 22959 |
| Grandparent’s health |  | 3.6 | 0.9 | 11261 |
| Conflict with children | High | 29.0 |  | 4236 |
|  | Low | 71.0 |  | 10376 |
| Conflict about grandchildren’s upbringing | High | 12.3 |  | 1750 |
|  | Low | 87.7 |  | 12423 |
| Savings (in euro) |  | 21766 | 92220 | 8828 |
| Grandparent’s education |  | 4.4 | 4.7 | 20985 |
| Grandparent employed | Yes | 31.1 |  | 5431 |
|  | No | 69.9 |  | 12623 |
| Grandparent has a partner | Yes | 62.7 |  | 14402 |
|  | No | 37.3 |  | 8564 |
| **Children** |  |  |  |  |
| Age of child |  | 36.4 | 9.9 | 22773 |
| Education of child |  | 5.7 | 4.7 | 21607 |
| Child employed | Yes | 78.3 |  | 17576 |
|  | No | 21.7 |  | 4859 |
| Child has a partner | Yes | 75.3 |  | 16254 |
|  | No | 24.7 |  | 5328 |
| **Grandchildren** |  |  |  |  |
| Age of youngest grandchild |  | 10.0 | 8.5 | 13797 |
| **Macro-economics** |  |  |  |  |
| Fertility rates^c^ |  | 1.6 | 0.22 | 22967 |
|  | Italy | 1.32 |  | 1946 |
|  | Greece | 1.33 |  | 1938 |
|  | Germany | 1.34 |  | 2268 |
|  | Spain | 1.34 |  | 2099 |
|  | Austria | 1.41 |  | 1713 |
|  | Switzerland | 1.42 |  | 688 |
|  | Netherlands | 1.71 |  | 2218 |
|  | Belgium | 1.72 |  | 3197 |
|  | Sweden | 1.77 |  | 2971 |
|  | Denmark | 1.80 |  | 1613 |
|  | France | 1.92 |  | 2299 |
| Regions | North/central | 63.5 |  | 14583 |
|  | South/central | 36.5 |  | 8384 |

^a^ percentage is shown for dichotomous variables

^b^ standard deviation is absent for dichotomous variables

^c^ standard deviation and range are absent for the country-specific fertility rates.

Are Participants who Report Almost Daily Grandparental Investment Substitute Parents?

SHARE does not provide information about whether grandparents are primary caretakers for grandchildren. If this were the case for grandparents looking after grandchildren on a daily basis, this level of investment could not be compared with other investment levels, where opportunity costs for grandparents occur. Therefore, we examined whether grandparents reporting almost daily investment actually looked after their grandchildren 24 hours a day. As displayed in Table S2, there is no indication that grandparents investing almost daily are primary caretakers. More than 90% of them reported looking after grandchildren for 10 hours a day or less.

**Table S2.** Cumulative percentage of hours of grandparental investment for the almost daily level

| **Hours looked after grandchildren almost daily** | **Biological grandparent *n* (%)** | **Non-biological grandparent *n* (%)** | **Total sample (%)** |
| --- | --- | --- | --- |
| 1 | 6.0 |  | 5.8 |
| 2 | 25.5 | 21.1 | 25.3 |
| 3 | 36.9 | 71.1 | 38.4 |
| 4 | 50.1 | 76.3 | 51.2 |
| 5 | 59.1 | 84.2 | 60.2 |
| 6 | 73.0 | 87.4 | 73.5 |
| 7 | 76.5 |  | 76.9 |
| 8 | 85.5 | 94.7 | 85.9 |
| 9 | 87.3 |  | 87.6 |
| 10 | 91.7 |  | 91.9 |
| 11 | 92.0 |  | 92.1 |
| 12 | 94.2 |  | 94.2 |
| 15 | 94.5 |  | 94.5 |
| 16 | 94.7 |  | 94.7 |
| 18 | 95.3 |  | 95.3 |
| 20 | 95.9 |  | 95.8 |
| 23 | 96.2 |  | 96.2 |
| 24 | 100.0 | 100.0 | 100.0 |

Identification of True Confounders

Only covariates showing significant variation in both grandparental characteristics and grandparental investment are considered to be true confounders. Table S3 presents the results of additional tests conducted to identify these true confounders. The *Biological relatedness* column shows results from the initial chi-square (χ^2^ for dichotomous variables) and Mann–Whitney U tests (*Z* values for continuous variables). Additionally, the *Investment* column presents Spearman coefficients (rho), indicating significant variation in investment levels for each covariate. The *True confounder* column shows whether or not a covariate was associated with both biological relatedness and grandparental investment: 17 variables were identified as true confounders and used in our main analysis predicting grandparental investment (Table 2 of our article and Table S6). They were biological relatedness, sex of grandparent, filial expectation, distance to (grand)child, number of children, number of grandchildren, age of grandparent, child, and grandchild, health status of grandparent, conflicts about the upbringing of grandchildren, education of grandparent and child, partner status of grandparent, work status of child, country-specific fertility rates, and regions. The covariate *Lineage* (and therefore sex of child), although not emerging as a true confounder, was also included in the final model. This variable’s lack of statistical association with biological relatedness is easily explained: a child’s sex cannot be expected to be dependent on whether or not the parent is a biological relative. However, there was a strong association with investment, as expected from a theoretical viewpoint. This important covariate was therefore also included in the multinomial logistic regression.

**Table S3.** List of true confounders associated with both biological relatedness and grandparental investment (chi-square, Mann–Whitney U tests, and Spearman correlations[*rho*])).

| **Variable** | **Biological relatednes**^a,b^ | **Grandparental investment** (*rho*) | **True confounder** |
| --- | --- | --- | --- |
| **Grandparents** |  |  |  |
| Investment | 127.4*** | ‑ | ‑ |
| Biological grandparent |  | .04** | Yes |
| Grandparent sex | 122.9*** | .02** | Yes |
| Grandparent lineage | 0.06 | .02** | No (Yes) |
| Filial expectations | -10.8*** | .07** | Yes |
| Distance to (grand)child | -13.9*** | -.14** | Yes |
| Number of children | -21.7*** | -.02** | Yes |
| Number of grandchildren | -1.9** | -.10** | Yes |
| Grandparent’s age | -22.5*** | -.37** | Yes |
| Grandparent’s health | -3.8*** | .12** | Yes |
| Conflict with children | 0.70 | .02** | No |
| Conflict about grandchildren’s upbringing | 18.9*** | .04** | Yes |
| Savings (in euro) | -0.98 | .02* | No |
| Grandparent’s education | -9.2*** | .10** | Yes |
| Grandparent employed | 0.38 | .00 | No |
| Grandparent has a partner | 250.3*** | .20** | Yes |
| **Children** |  |  |  |
| Age of child | -18.8*** | -.35** | Yes |
| Education of child | -5.8*** | .11** | Yes |
| Child employed | 41.4*** | .05** | Yes |
| Child has a partner | 1.1 | -.02** | No |
| **Grandchildren** |  |  |  |
| Age of youngest grandchild | -4.5*** | -.43** | Yes |
| **Macro-economics** |  |  |  |
| Fertility rates | -20.4*** | -.02** | Yes |
| Regions | 602.0*** | .02** | Yes |

^a^ *χ^2^* values are given for dichotomous variables

^b^ *Z* values are given for continuous variables

* *p* < .05. ** *p* < .01. *** *p* < .001.

Potential Mediation Effect of Age on Health

We conducted an additional multinomial logistic regression to investigate whether the effect of grandparental age on grandparental investment was mediated by grandparental health (Table S4). Results show that there was no mediation of age through health. In fact, both variables independently accounted for variance in each of the investment levels relative to *no investment* (reference level).

**Table S4.** Odds ratios (Exp[B]) and significance levels of multinomial logistic regression testing the potential mediation effect of grandparent’s age by health on grandparental investment.

|  | **Almost daily childcare** | | **Almost weekly childcare** | | **Almost monthly childcare** | | **Less often childcare** | |
| --- | --- | --- | --- | --- | --- | --- | --- | --- |
|  | **Exp(B)** | ***p*** | **Exp(B)** | ***p*** | **Exp(B)** | ***p*** | **Exp(B)** | ***p*** |
| Grandparent’s age | .92 | *** | .90 | *** | .91 | *** | .92 | *** |
| Grandparent’s health | .87 | ** | 1.39 | *** | 1.34 | *** | 1.37 | *** |

* *p* < .05. ** *p* < .01. *** *p* < .001.

Can Grandparental Investment be Predicted by Fertility Rates or Geographic Regions?

Using the SHARE database, Hank and Buber [36] found a north–south gradient in regular grandparental investment across Europe. They showed that grandparents in the Nordic countries looked after their grandchildren less regularly than did those in central and southern Europe. This difference could be attributable to macro-economic determinants, such as the pattern of childcare provided by the state probably leading to higher fertility rates but less grandparental involvement in the Nordic countries. Table 1 of our article shows that being a non-biological grandparent is associated with higher fertility rates, with living in the north/central region of Europe, and also with less frequent investment. Table S3 shows that higher fertility rates (e.g., in the Nordic countries) are also associated with less frequent grandparental investment. Therefore, fertility rates may reflect underlying structural determinants for each country [3], and both variables should be taken into account. In order to test the effect of fertility rates versus geographic regions, we included both variables in a multinomial logistic regression (Table S5).

Results reveal that both variables independently account for variance in almost all of the investment levels relative to *no investment* (reference level).

From a theoretical viewpoint, fertility rates may reflect macroeconomic structures in a more tangible way than plain geographic regions. However, the independent influence of geographic borders indicates that there may be socio-economical differences strongly shaping grandparental investment. Shedding light on these influences in more detail would go far beyond the scope of this study. However, including both variables in the main analysis (Table 2 of the article, and Table S6 below) can provide some evidence for possible socio-economic influences that warrant further investigation in future studies.

**Table S5.** Odds ratios (Exp[B]) and significance levels of multinomial logistic regression testing the independent effects of fertility rates and regions of Europe.

|  | **Almost daily childcare** | | **Almost weekly childcare** | | **Almost monthly childcare** | | **Less often childcare** | |
| --- | --- | --- | --- | --- | --- | --- | --- | --- |
|  | **Exp(B)** | ***p*** | **Exp(B)** | ***p*** | **Exp(B)** | ***p*** | **Exp(B)** | ***p*** |
| Fertility rates^c^ | .06 | *** | .73 | * | 2.55 | *** | 5.68 | *** |
| Regions | .85 | * | 1.75 | *** | 1.72 | *** | 1.37 | *** |

* *p* < .05. ** *p* < .01. *** *p* < .001.

Details of the Main Multinomial Logistic Regression

Table S6 provides more details of the multinomial regression for each investment level. The covariates are ordered by the strength of their odds ratio and significance level, making it easier for readers to identify which covariates are most important for which level of investment.

**Table S6.** Detailed results for each investment level from the multinomial logistic regression investigating grandparental investment.

| **Almost daily investment** | **Exp(B)** | ***p*** | **95% CI Exp(B)** | | **B** | **S.E.** | **Wald** |
| --- | --- | --- | --- | --- | --- | --- | --- |
| Child employed (yes) | 1.95 | *** | 1.36 | 2.79 | 0.67 | 0.18 | 13.21 |
| Fertility rates | 0.13 | ** | 0.03 | 0.58 | -2.02 | 0.75 | 7.27 |
| Filial expectations | 1.79 | *** | 1.47 | 2.18 | 0.58 | 0.10 | 33.79 |
| Grandparent has a partner (yes) | 1.79 | *** | 1.29 | 2.49 | 0.58 | 0.17 | 12.16 |
| Regions (north/central) | 0.43 | ** | 0.25 | 0.74 | -0.85 | 0.28 | 9.32 |
| Grandparent lineage (maternal) | 1.54 | ** | 1.16 | 2.02 | 0.47 | 0.14 | 9.27 |
| Biological grandparent (yes) | 1.51 | * | 0.81 | 2.82 | 0.41 | 0.32 | 1.66 |
| Distance to (grand)child | 0.71 | *** | 0.65 | 0.77 | -0.35 | 0.04 | 64.61 |
| Number of children | 0.71 | ** | 0.57 | 0.88 | -0.34 | 0.11 | 9.39 |
| Grandparent’s health | 0.83 | * | 0.71 | 0.98 | -0.18 | 0.08 | 4.75 |
| Age of youngest grandchild | 0.91 | *** | 0.89 | 0.94 | -0.09 | 0.02 | 33.57 |
| Education of child | 1.09 | ** | 1.02 | 1.16 | 0.08 | 0.03 | 6.14 |
| Grandparent’s age | 0.92 | *** | 0.89 | 0.95 | -0.08 | 0.02 | 31.95 |
| Number of grandchildren | 1.08 | * | 1.00 | 1.16 | 0.07 | 0.04 | 3.68 |
| Age of child | 0.93 | *** | 0.90 | 0.97 | -0.07 | 0.02 | 11.79 |
| Household identifier | 0.97 |  | 0.95 | 1.02 | -0.04 | 0.01 | 6.22 |
| Grandparent sex (female) | 1.24 |  | 0.91 | 1.69 | 0.21 | 0.16 | 1.79 |
| Conflict about grandchildren’s upbringing (high) | 1.18 |  | 0.83 | 1.68 | 0.17 | 0.18 | 0.85 |
| Grandparent’s education | 1.01 |  | 0.95 | 1.09 | 0.01 | 0.03 | 0.17 |
| **Almost weekly investment** | **Exp(B)** |  | **95% CI Exp(B)** | | **B** | **S.E** | **Wald** |
| Biological grandparent (yes) | 1.57 | * | 1.09 | 2.25 | 0.45 | 0.19 | 5.76 |
| Grandparent has a partner (yes) | 1.38 | ** | 1.09 | 1.75 | 0.32 | 0.12 | 7.07 |
| Filial expectations | 1.24 | ** | 1.08 | 1.41 | 0.21 | 0.07 | 9.56 |
| Distance to (grand)child | 0.79 | *** | 0.74 | 0.84 | -0.24 | 0.03 | 53.09 |
| Grandparent’s health | 1.18 | ** | 1.04 | 1.33 | 0.16 | 0.06 | 6.91 |
| Grandparent’s education | 1.09 | *** | 1.03 | 1.15 | 0.09 | 0.03 | 11.93 |
| Age of youngest grandchild | 0.92 | *** | 0.90 | 0.94 | -0.08 | 0.01 | 45.45 |
| Child employed (yes) | 1.08 | ns | 0.83 | 1.40 | 0.07 | 0.13 | 0.30 |
| Grandparent’s age | 0.93 | *** | 0.91 | 0.95 | -0.08 | 0.01 | 47.43 |
| Age of child | 0.94 | *** | 0.91 | 0.96 | -0.07 | 0.02 | 18.86 |
| Grandparent sex (female) | 1.22 |  | 0.97 | 1.54 | 0.20 | 0.12 | 2.84 |
| Fertility rates | 0.81 |  | 0.32 | 1.64 | -0.21 | 0.42 | 0.25 |
| Regions (north/central) | 1.13 |  | 0.80 | 1.71 | 0.12 | 0.18 | 0.46 |
| Conflict about grandchildren’s upbringing (high) | 0.86 |  | 0.63 | 1.17 | -0.15 | 0.16 | 0.92 |
| Grandparent lineage (maternal) | 1.06 |  | 0.86 | 1.30 | 0.06 | 0.11 | 0.30 |
| Number of grandchildren | 1.04 |  | 0.98 | 1.09 | 0.04 | 0.03 | 1.62 |
| Number of children | 0.97 |  | 0.83 | 1.14 | -0.03 | 0.08 | 0.10 |
| Education of child | 1.00 |  | 0.95 | 1.06 | 0.01 | 0.03 | 0.01 |
| Household identifier | 0.96 |  | 0.95 | 1.03 | -0.02 | 0.01 | 2.59 |
| **Almost monthly investment** | **Exp(B)** |  | **95% CI Exp(B)** | | **B** | **S.E.** | **Wald** |
| Fertility rates | 4.02 | ** | 1.35 | 7.90 | 1.39 | 0.46 | 9.03 |
| Regions (north/central) | 1.66 | * | 1.08 | 2.56 | 0.51 | 0.22 | 5.28 |
| Filial expectations | 1.46 | *** | 1.24 | 1.71 | 0.38 | 0.08 | 21.47 |
| Child employed (yes) | 1.37 | * | 1.00 | 1.91 | 0.32 | 0.17 | 3.64 |
| Grandparent sex (female) | 1.31 | * | 1.00 | 1.70 | 0.27 | 0.14 | 3.83 |
| Age of youngest grandchild | 0.90 | *** | 0.87 | 0.92 | -0.11 | 0.02 | 50.78 |
| Grandparent’s education | 1.09 | ** | 1.02 | 1.15 | 0.08 | 0.03 | 7.25 |
| Grandparent’s age | 0.93 | *** | 0.91 | 0.96 | -0.07 | 0.01 | 29.74 |
| Education of child | 0.93 | * | 0.88 | 1.00 | -0.07 | 0.03 | 4.08 |
| Age of child | 0.97 | * | 0.93 | 1.00 | -0.04 | 0.02 | 3.82 |
| Household identifier | 0.99 |  | 0.96 | 1.04 | -0.03 | 0.02 | 3.77 |
| Grandparent lineage (maternal) | 0.83 |  | 0.65 | 1.05 | -0.19 | 0.12 | 2.39 |
| Grandparent has a partner (yes) | 1.14 |  | 0.87 | 1.50 | 0.13 | 0.14 | .86 |
| Conflict about grandchildren’s upbringing (high) | 0.87 |  | 0.59 | 1.29 | -0.14 | 0.20 | 0.46 |
| Number of children | 1.11 |  | 0.92 | 1.33 | 0.10 | 0.10 | 1.14 |
| Grandparent’s health | 1.10 |  | 0.96 | 1.26 | 0.09 | 0.07 | 1.78 |
| Biological grandparent (yes) | 0.98 |  | 0.68 | 1.42 | -0.02 | 0.19 | 0.01 |
| Distance to (grand)child | 0.98 |  | 0.92 | 1.06 | -0.02 | 0.04 | 0.19 |
| Number of grandchildren | 1.00 |  | 0.94 | 1.06 | 0.00 | 0.03 | 0.01 |
| **Less often investment** | **Exp(B)** |  | **95% CI Exp(B)** | | **B** | **S.E.** | **Wald** |
| Fertility rates | 5.41 | *** | 2.34 | 12.50 | 1.61 | 0.43 | 15.63 |
| Grandparent sex (female) | 1.29 | * | 1.03 | 1.62 | 0.25 | 0.12 | 4.80 |
| Grandparent’s health | 1.23 | *** | 1.10 | 1.39 | 0.21 | 0.06 | 11.88 |
| Number of children | 1.20 | * | 1.03 | 1.40 | 0.18 | 0.08 | 5.14 |
| Distance to (grand)child | 1.14 | *** | 1.07 | 1.21 | 0.13 | 0.03 | 17.35 |
| Age of youngest grandchild | 0.93 | *** | 0.91 | 0.95 | -0.07 | 0.01 | 44.83 |
| Grandparent’s age | 0.96 | *** | 0.94 | 0.98 | -0.04 | 0.01 | 15.53 |
| Age of child | 0.97 | * | 0.94 | 1.00 | -0.03 | 0.01 | 4.89 |
| Conflict about grandchildren’s upbringing (high) | 0.84 |  | 0.60 | 1.17 | -0.18 | 0.17 | 1.08 |
| Biological grandparent (yes) | 1.10 |  | 0.79 | 1.53 | 0.10 | 0.17 | 0.31 |
| Filial expectations | 1.09 |  | 0.96 | 1.24 | 0.09 | 0.07 | 1.78 |
| Grandparent lineage (maternal) | 1.07 |  | 0.87 | 1.30 | 0.06 | 0.10 | 0.37 |
| Grandparent has a partner (yes) | 0.93 |  | 0.75 | 1.17 | -0.07 | 0.12 | 0.35 |
| Child employed (yes) | 1.05 |  | 0.80 | 1.36 | 0.05 | 0.14 | 0.12 |
| Grandparent’s education | 1.05 |  | 0.99 | 1.10 | 0.04 | 0.03 | 2.99 |
| Regions (north/central) | 1.03 |  | 0.70 | 1.50 | 0.03 | 0.19 | 0.02 |
| Education of child | 0.97 |  | 0.92 | 1.03 | -0.03 | 0.03 | 1.48 |
| Number of grandchildren | 0.98 |  | 0.94 | 1.04 | -0.02 | 0.03 | 0.37 |
| Household identifier | 1.00 |  | 0.95 | 1.04 | -0.02 | 0.01 | 1.12 |

* *p* < .05. ** *p* < .01. *** *p* < .001.

Testing the Robustness of the Results

To test the robustness of the initial multinomial logistic regression predicting grandparental investment, we re-ran the analyses using different methods and altering the outcome variable. The covariates included remained the same as in the initial analysis.

First, we ran a binary logistic regression with the outcome variable dichotomized into high (almost daily/weekly) and low investment (almost monthly/less often/never). The results (Table S7) are similar to those produced by the initial multinomial logistic regression. Second, all non-investors—who accounted for 50.3% of the total sample—were excluded, and a multinomial logistic regression (Table S8) and a binary logistic regression (Table S9) were conducted. Again, the results were very similar to the initial analysis, suggesting that these are robust effects.

**Table S7.** Odds ratios (Exp[B]), significance levels, and confidence intervals of binary logistic regression for entire sample including non-investors (reference category: low investment).

| **Independent variables** | **Exp(B)** | ***p*** | **95% CI Exp(B)** | |
| --- | --- | --- | --- | --- |
| Biological grandparent (yes) | 1.50 | ** | 1.11 | 2.04 |
| Grandparent sex (female) | 1.09 |  | 0.90 | 1.32 |
| Grandparent lineage (maternal) | 1.22 | * | 1.03 | 1.44 |
| Filial expectations | 1.26 | *** | 1.13 | 1.41 |
| Distance to (grand)child | 0.74 | *** | 0.70 | 0.78 |
| Number of children | 0.84 | ** | 0.74 | 0.96 |
| Number of grandchildren | 1.06 | ** | 1.01 | 1.10 |
| Grandparent’s age | 0.95 | *** | 0.93 | 0.96 |
| Grandparent’s health | 1.00 |  | 0.91 | 1.11 |
| Conflict about grandchildren’s upbringing (high) | 1.03 |  | 0.81 | 1.32 |
| Grandparent’s education | 1.04 |  | 1.00 | 1.08 |
| Grandparent has a partner (yes) | 1.50 | *** | 1.23 | 1.82 |
| Age of child | 0.95 | *** | 0.93 | 0.98 |
| Education of child | 1.05 | * | 1.01 | 1.09 |
| Child employed (yes) | 1.22 |  | 0.98 | 1.51 |
| Age of youngest grandchild | 0.94 | *** | 0.92 | 0.96 |
| Fertility rates | 0.22 | *** | 0.11 | 0.45 |
| Regions (north/central) | 0.77 | * | 0.57 | 1.03 |
| Household identifier | 0.99 |  | 0.95 | 1.02 |

* *p* < .05. ** *p* < .01. *** *p* < .001.

**Table S8.** Odds ratios (Exp[B]) and significance levels of a multinomial logistic regression for each grandparental investment level excluding non-investors (reference category: less often).

|  | **Almost daily childcare** | | **Almost weekly childcare** | | **Almost monthly childcare** | |
| --- | --- | --- | --- | --- | --- | --- |
| **Independent variables** | **Exp(B)** | ***p*** | **Exp(B)** | ***p*** | **Exp(B)** | ***p*** |
| Biological grandparent (yes) | 1.50 | * | 1.59 | * | 0.96 |  |
| Grandparent sex (female) | 0.94 |  | 0.92 |  | 0.96 |  |
| Grandparent lineage (maternal) | 1.32 | * | 0.98 |  | 0.78 |  |
| Filial expectations | 1.64 | *** | 1.15 |  | 1.38 | *** |
| Distance to (grand)child | 0.61 | *** | 0.68 | *** | 0.87 | ** |
| Number of children | 0.67 | ** | 0.89 |  | 0.95 |  |
| Number of grandchildren | 1.08 | * | 1.04 |  | 1.00 |  |
| Grandparent’s age | 0.95 | ** | 0.96 | ** | 0.97 | * |
| Grandparent’s health | 0.64 | *** | 0.94 |  | 0.91 |  |
| Conflict about grandchildren’s upbringing (high) | 1.51 |  | 1.11 |  | 1.06 |  |
| Grandparent’s education | 0.97 |  | 1.04 |  | 1.04 |  |
| Grandparent has a partner (yes) | 2.13 | *** | 1.50 | ** | 1.22 |  |
| Age of child | 0.97 |  | 0.97 | * | 0.99 |  |
| Education of child | 1.13 | ** | 1.03 |  | 0.96 |  |
| Child employed (yes) | 1.66 | * | 0.93 |  | 1.23 |  |
| Age of youngest grandchild | 0.97 |  | 0.99 |  | 0.97 | * |
| Fertility rates | 0.03 | *** | 0.15 | *** | 0.81 |  |
| Regions (north/central) | 0.54 | *** | 1.00 |  | 1.44 |  |
| Household identifier | 0.98 |  | 0.95 |  | 0.97 |  |

* *p* < .05. ** *p* < .01. *** *p* < .001.

**Table S9.** Odds ratios (Exp[B]), significance levels, and confidence intervals of binary logistic regression excluding non-investors (reference category: low investment).

| **Independent variables** | **Exp(B)** | ***p*** | **95% CI Exp(B)** | |
| --- | --- | --- | --- | --- |
| Biological grandparent (yes) | 1.62 | * | 1.08 | 2.47 |
| Grandparent sex (female) | 1.25 | * | 1.00 | 1.57 |
| Grandparent lineage (maternal) | 1.88 | *** | 1.53 | 2.32 |
| Filial expectations | 1.29 | *** | 1.12 | 1.48 |
| Distance to (grand)child | 0.63 | *** | 0.59 | 0.67 |
| Number of children | 0.46 | *** | 0.39 | 0.54 |
| Number of grandchildren | 1.09 | ** | 1.03 | 1.15 |
| Grandparent’s age | 0.96 | *** | 0.94 | 0.98 |
| Grandparent’s health | 0.99 |  | 0.87 | 1.11 |
| Conflict about grandchildren’s upbringing (high) | 0.89 |  | 0.66 | 1.18 |
| Grandparent’s education | 1.08 | ** | 1.03 | 1.13 |
| Grandparent has a partner (yes) | 1.70 | *** | 1.34 | 2.17 |
| Age of child | 0.96 | * | 0.94 | 0.99 |
| Education of child | 0.99 |  | 0.95 | 1.05 |
| Child employed (yes) | 1.49 | ** | 1.15 | 1.93 |
| Age of youngest grandchild | 0.93 | *** | 0.91 | 0.96 |
| Fertility rates | 0.53 |  | 0.22 | 1.27 |
| Regions (north/central) | 0.76 |  | 0.53 | 1.10 |
| Household identifier | 0.98 |  | 0.96 | 1.03 |

* *p* < .05. ** *p* < .01. *** *p* < .001.
